# Interventions to improve the outcomes of frail people having surgery: A systematic review

**DOI:** 10.1371/journal.pone.0190071

**Published:** 2017-12-29

**Authors:** Daniel I. McIsaac, Tim Jen, Nikhile Mookerji, Abhilasha Patel, Manoj M. Lalu

**Affiliations:** 1 Department of Anesthesiology and Pain Medicine, University of Ottawa, Ottawa, Ontario, Canada; 2 Department of Anesthesiology and Pain Medicine, The Ottawa Hospital, Ottawa, Ontario, Canada; 3 Clinical Epidemiology Program, Ottawa Hospital Research Institute, Ottawa, Ontario, Canada; 4 Faculty of Medicine, University of Ottawa, Ottawa, Ontario, Canada; University of Glasgow, UNITED KINGDOM

## Abstract

**Background:**

Frailty is an important prognostic factor for adverse outcomes and increased resource use in the growing population of older surgical patients. We identified and appraised studies that tested interventions in populations of frail surgical patients to improve perioperative outcomes.

**Methods:**

We systematically searched Cochrane, CINAHL, EMBASE and Medline to identify studies that tested interventions in populations of frail patients having surgery. All phases of study selection, data extraction, and risk of bias assessment were done in duplicate. Results were synthesized qualitatively per a prespecified protocol (CRD42016039909).

**Results:**

We identified 2 593 titles; 11 were included for final analysis, representing 1 668 participants in orthopedic, general, cardiac, and mixed surgical populations. Only one study was multicenter and risk of bias was moderate to high in all studies. Interventions were applied pre- and postoperatively, and included exercise therapy (n = 4), multicomponent geriatric care protocols (n = 5), and blood transfusion triggers (n = 1); no specific surgical techniques were compared. Exercise therapy, applied pre-, or post-operatively, was associated with significant improvements in functional outcomes and improved quality of life. Multicomponent protocols suffered from poor compliance and difficulties in implementation. Transfusion triggers had no significant impact on mortality or other outcomes.

**Conclusions:**

Despite a growing literature that demonstrates strong independent associations between frailty and adverse outcomes, few interventions have been tested to improve the outcomes of frail surgical patients, and most available studies are at substantial risk of bias. Multicenter, low risk of bias, studies of perioperative exercise are needed, while substantial efforts are required to develop and test other interventions to improve the outcomes of frail people having surgery.

## Introduction

Western populations are aging rapidly.[1,2] Older people have surgery at over two times the rate of younger individuals,[3] and advanced age is a well-established risk factor for adverse postoperative outcomes.[4,5] However, amongst the older surgical population, outcomes vary substantially.[6] Frailty, a state of increased vulnerability to stressors due to age-, and disease-related deficits that accumulate across multiple domains, is a key factor in explaining the increased rates of complications, healthcare resource use, loss of independence, and mortality experienced by older surgical patients[7–11]

The prevalence of frailty increases exponentially with age.[12] Therefore, as our population ages, an increasing number of frail patients are expected to present for surgery. In fact, contemporary studies estimate that 25–40% of older patients having major surgery are frail or pre-frail.[13–15] Based on a conservative estimate that frailty is associated with a 2- to 3-fold increase in the relative risk of adverse postoperative events,[8] we estimate that the proportion of adverse events attributable[16] to frailty is 25–50%. However, despite the strong and increasingly well-recognized association of frailty with adverse postoperative events and increased resource use across surgical specialties,[8,9,17] and the multitude of instruments that have been used to diagnose frailty,[18] interventions specifically tailored to frail surgical patients are not commonly described in the literature, and have not been systematically reviewed. Knowledge generated from such a synthesis is needed to inform current care and future research. Therefore, we undertook a systematic review to identify interventions that have been tested in populations of frail surgical populations to improve health outcomes, patient experience or costs of care.[19]

## Materials and methods

This systematic review was performed in accordance with guidelines from the Cochrane Collaboration,[20] and is reported according to the Preferred Reporting Items for Systematic Reviews and Meta-Analyses guidelines (see checklist in S1 File).[21] The study protocol was registered with the International Prospective Register of Systematic Reviews (2016:CRD42016039909).

### Search strategy

A systematic search strategy was designed in consultation with an information specialist, and then reviewed and finalized using the peer review of electronic search strategy checklist.[22] The search strategy is provided in Table A in S2 File. We employed a broad strategy using keywords and controlled vocabulary to identify frailty and surgical procedures. The search did not place limitations on outcomes or study designs. No language restrictions were applied, and all databases (Cochrane, Medline, Cumulative Index of Nursing and Allied Health Literature, and the Excerpta Medica Database) were searched from inception to February 14, 2016. Grey literature was searched and considered, including conference proceedings (2010–2016) from the American College of Surgeons, American Geriatrics Society, American Society of Anesthesiology, British Geriatrics Society, and the European Geriatrics Society, as well as conference abstracts identified through our database searches. We also searched ClinicalTrials.gov to identify planned, in-progress or completed studies that had not yet been reported.

### Inclusion and exclusion criteria

Randomized and non-randomized (e.g., cohort, controlled before after, interrupted time series, other quasi-randomized designs) studies were eligible for inclusion, however, non-experimental studies (such as case reports or case series) were excluded. To be included, studies had to evaluate a population of frail individuals having surgery (endovascular cardiac valve procedures, endoscopic procedures, and cataract surgery were not included as perioperative processes and trajectories were felt to differ substantially from prototypical surgical procedures), or have a specific subgroup of frail patients where frailty-specific intervention and outcome data could be extracted. In the case of a mixed population (i.e., surgical and medical), surgical patients had to represent the majority of included participants. Included studies had to state the specific method used to define individuals as frail, however, we placed no limitations on what frailty definitions were acceptable. Studies could test any intervention, so long as it was applied in the perioperative period and was related to the fact that patients were having, or had surgery. We did not limit inclusion to specific outcome types, however, we did categorize outcomes in one of the three domains of the IHI Triple Aim outcome framework (health, cost, experience).[19]

### Selection of included studies and data extraction

All identified titles and abstracts, and conference proceedings were screened in duplicate by two independent reviewers. When adherence to inclusion/exclusion criteria was unclear, studies were moved forward for full text review. Full text review was also performed in duplicate, and disagreement at any stage was resolved in discussion with the primary investigator (DM). The reference lists of all included articles were searched to identify any other studies that may have been missed by our search strategy.

For data collection, a form designed specifically for this review was first piloted on six studies, and then applied to all studies. Data was extracted in duplicate, and reviewed in a triad that included both reviewers and the primary investigator. Publication characteristics, patient and surgical factors, details of the intervention, and study outcomes were extracted for all included studies. All citation screening, full text review, and data collection was performed using DistillerSR® (Evidence Partners, Ottawa, Canada).

### Risk of bias assessment

Risk of bias assessments were conducted for all studies. Non-randomized studies were assessed using the Risk of Bias in Non-randomized studies of Interventions (ROBINS-I);[23] randomized controlled trials (RCTs) were assessed with the Cochrane Risk of Bias Tool for randomized trials.[20] The scales for each risk of bias tool were modified to provide consistent scoring across study designs. All risk of bias assessments were done in duplicate by the primary investigator and a second team member; disagreements were resolved by consensus.

### Analysis and data synthesis

We summarized the study designs, frailty instruments, surgeries, patient characteristics, intervention characteristics and outcomes reported. We did not anticipate identifying adequately homogenous data to support formal meta-analysis, and we therefore pre-specified a qualitative approach to data synthesis. We organized our qualitative synthesis first around the type of intervention, then by surgical population, and finally be phase of the perioperative period where intervention was employed. We also synthesized the types of outcomes that were studied within these groupings.

## Results

**Fig 1:**
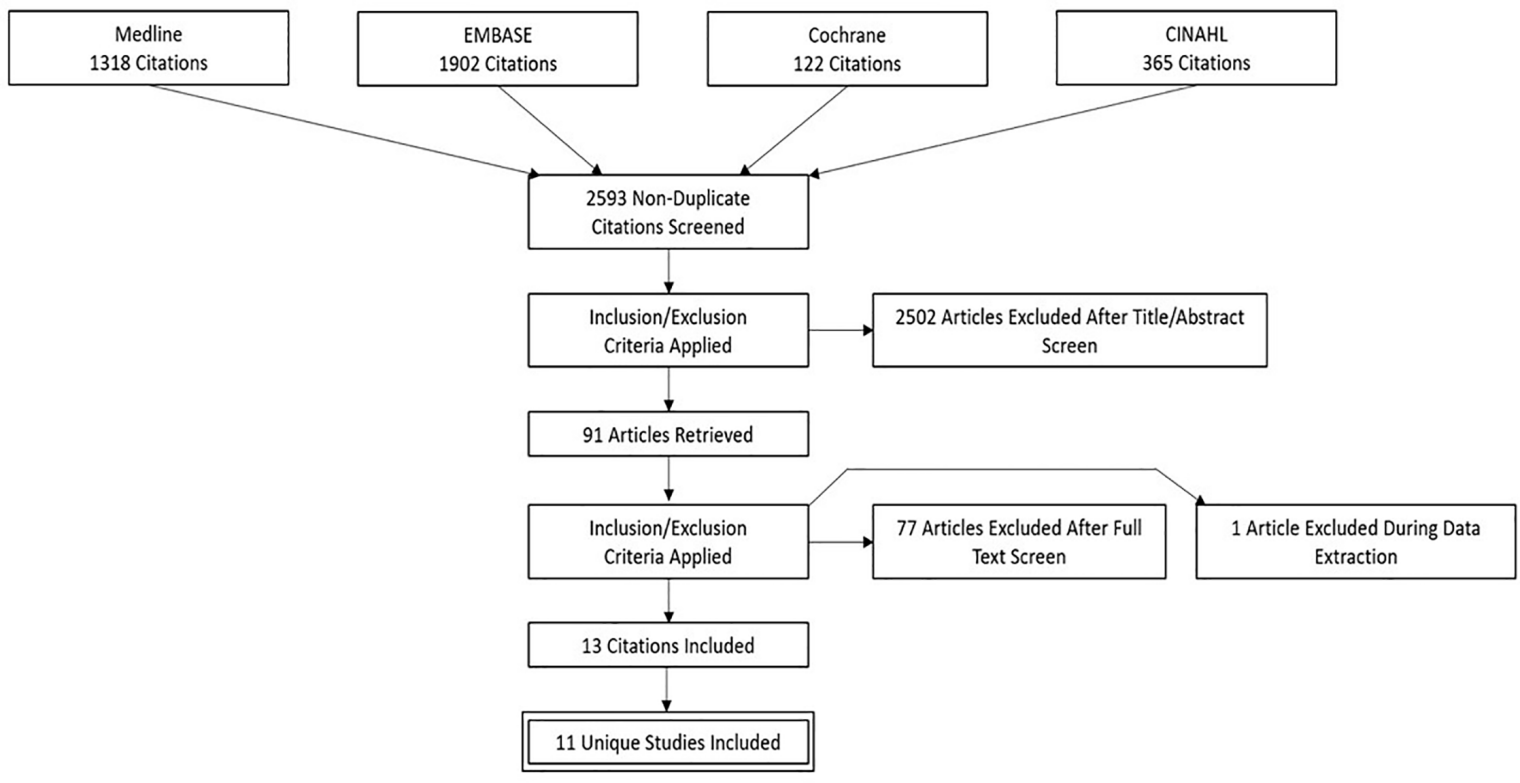
Flow diagram outlining selection of studies.

Following removal of duplicate records, we identified 2 593 unique title and abstracts to review, and as described in Fig 1, included 11 studies for final analysis (1 study generated 3 unique citations the result of which were considered together as a single study). The one conference abstract identified was not included in our formal synthesis, as frailty definitions used were not described, and because inadequate information was available to assess risk of bias. Seven trials were identified through ClinicalTrials.gov (November 23^rd^, 2016); one had completed recruitment (an email to the investigators requesting data was not returned), four were currently recruiting, and two were not yet open for recruitment. The conference abstract and summaries of ClincalTrials.gov protocols are provided in Table B in S2 File.

### Study and population characteristics

Six of the included studies were RCTs, and five were observational (four controlled before after, and one whose design was unclear but which appeared to be most consistent with a prospective non-randomized trial;[24] Table 1). Sample sizes ranged from 21 to 386 participants (1 668 total). Mean participant age was older than 70 years in all studies. Surgery types included general surgery (three studies), cardiac (two studies), orthopedic (four studies), solid tumor (one study) and mixed (one study). Surgical urgency included elective (six studies), emergency (two studies), mixed (one study), and not reported (two studies). Other details are provided in Table 1. Frailty was defined by geriatric assessment in three studies, the Identification of Seniors at Risk questionnaire in two studies, Fried’s Frailty Phenotype in one study, Groningen Frailty Indicator (GFI) for one study, Clinical Frailty Scale for one study, and physical performance measures in three studies.

**Table 1:** Characteristics of included studies.

Source	Study Type	Surgery	Frailty Instrument	Control (n)	Intervention (n)	Mean age	Intervention
Bakker et al, 2014^27^	CBA	Mixed	Geriatric examination	191	195	77	Enhanced care protocol
Binder et al, 2004^33^	RCT	Hip Fracture	mPPT score and ADLs	44	46	80	Post-operative exercise
Chen et al, 2014^26^	CBA	General	Fried’s frailty phenotype	52	52	73	Enhanced care protocol
Gorelik et al, 2015^24^	Unclear	General	Geriatric examination	35	36	82	Enhanced care protocol
Gregersen et al, 2015^35−37^	RCT	Hip Fracture	Comprehensive Geriatric Assessment	140	144	86	Blood transfusion trigger
Hempenius et al, 2013^29^	RCT	Solid tumor	Groningen Frailty Indicator	149	148	77	Enhanced care protocol
Hoogeboom et al, 2010^30^	RCT	Hip replacement	Clinical Frailty Scale	11	10	77	Pre-operative exercise
Indrakusuma et al, 2014^25^	CBA	General	ISAR	50	50	81	Enhanced care protocol
Molino-Lova et al, 2011^34^	RCT	Cardiac	SPPB score	48	51	75	Post-operative exercise
Oosting et al, 2012^31^	RCT	Hip replacement	ISAR	15	15	77	Pre-operative exercise
Opasich et al, 2010^32^	CBA	Cardiac	BPOMA	74	150	75	Post-operative exercise

BPOMA: Balance Performance Oriented Mobility Assessment; CBA: controlled before after; ISAR: Identification of Seniors At Risk; mPPT: modified version of the Physical Performance Test; RCT: randomized controlled study; SPPB: Short Physical Performance Battery score

### Intervention characteristics

**Fig 2:**
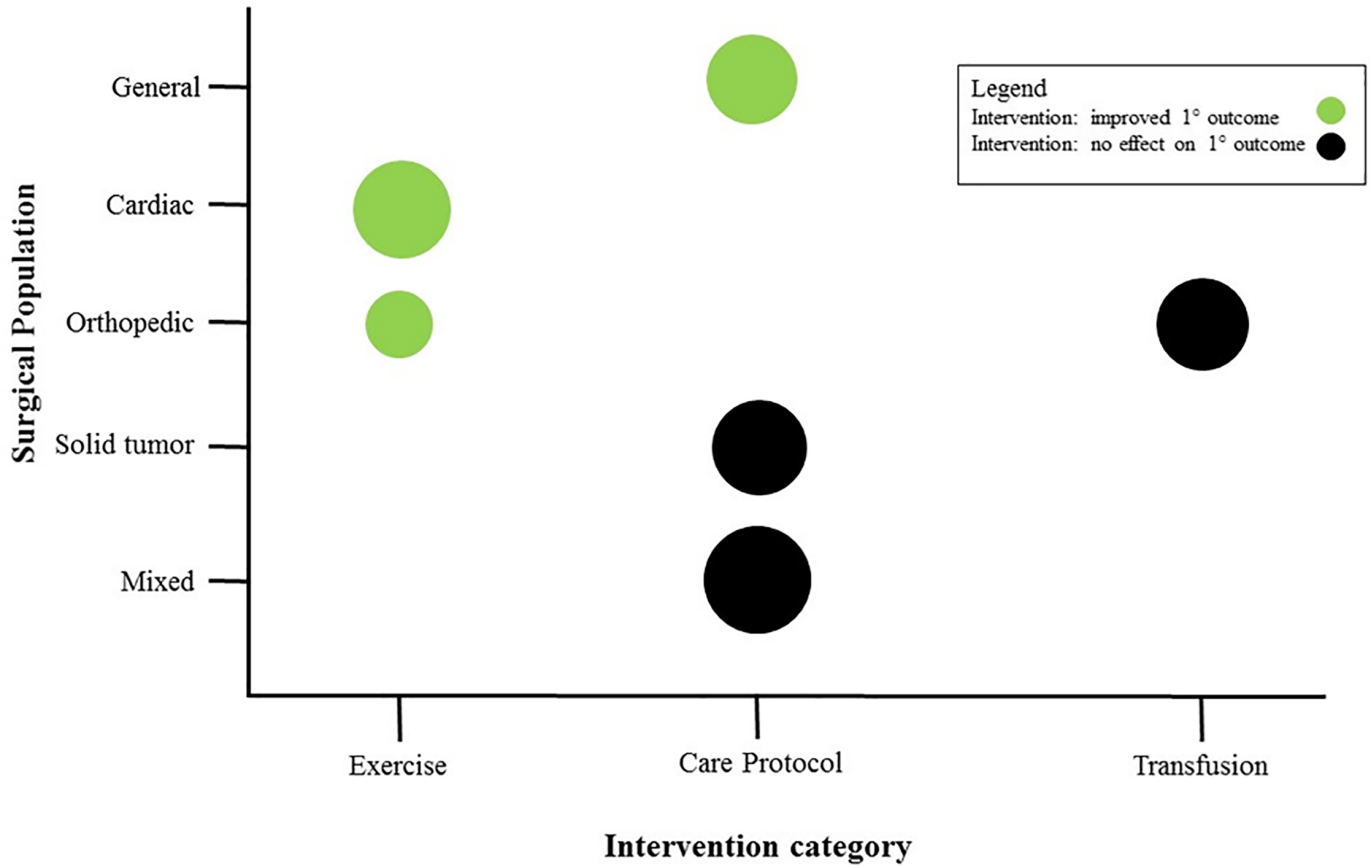
Summary of study outcomes by intervention type and surgical population. The size of each circle is proportional to the number of participants in each grouping.

Interventions were applied in the pre- and postoperative period; however, no specific intraoperative interventions were identified. Three categories of interventions were identified: multicomponent geriatric care protocols (n = 5), exercise interventions (n = 5), and transfusion triggers (n = 1). Specific details for each intervention are provided in Table 2, while trends in outcome effects across intervention types and surgical populations are described in Fig 2.

**Table 2:** Description of interventions and outcomes.

Source	Intervention Timing	Intervention	Control Group Intervention	Outcome(s)	Outcome Window	Result
Bakker, 2014^27^	Pre & Post	Care Protocol:	Standard care	**Hospital-acquired delirium**	In-hospital	No difference
		Orientation		**Cognitive decline**	In-hospital	No difference
		Mobilization		**Physical decline**	At discharge	**Worse with intervention group**
		Day program activities		**ADL**	At discharge	**Worse with intervention**
		Physiotherapy consult		ADL	At discharge	No difference
		Dietitian consult		ADL	3 months post-discharge	**Better with intervention**
		Discharge planning		Readmission	30 days post-discharge	No difference
		Medication review		Unplanned readmission	30 days post-discharge	No difference
		CGA by geriatrician		Caregiver burden	3 months post-discharge	No difference
Binder, 2004^33^	Post	Exercise	Non-personalized exercise without weight training	**Modified Physical Performance Test**	6 months after surgery	**Better with intervention**
				**Functional Status Questionnaire**	6 months after surgery	**Better with intervention**
				**Basic ADL**	6 months after surgery	No difference
				**Instrumental ADL**	6 months after surgery	No difference
				**Assistive devices use**	6 months after surgery	**Less use with intervention**
				Knee extension strength	6 months after surgery	**Better with intervention**
				Walking speed	6 months after surgery	**Better with intervention**
				Single limb stance time	6 months after surgery	**Better with intervention**
				Berg balance score	6 months after surgery	**Better with intervention**
				Total fat-free mass	6 months after surgery	No difference
				Bone mineral density	6 months after surgery	No difference
				SF-36 score	6 months after surgery	**Better health, physicial and social function with intervention**
				Hip Rating Questionnaire	6 months after surgery	**Better with intervention**
Chen, 2014^26^	Post	Care Protocol:	Standard care	*Frailty rate	At discharge	**Better with intervention**
					3 months post-discharge	No difference
		Early mobilization		Transitions between frailty states	From admission to discharge	**Better with intervention**
		Oral and nutritional assistance				
		Orientating communication			From admission to 3-months post-discharge	No difference
Gorelik, 2015^24^	Post	Care Protocol:	Standard care	*Stability	6 months after surgery	**Better with intervention**
				Walking	6 months after surgery	**Better with intervention**
		Rehabilitation		Malnutrition	6 months after surgery	**Better with intervention**
		Nutrition support		Cognitive disorders	6 months after surgery	**Better with intervention**
		Psychotherapy		Moral status	6 months after surgery	**Better with intervention**
		Home care for some		Independence	6 months after surgery	**Better with intervention**
Gregersen, 2015^35−37^	Post	Restrictive blood transfusion	Liberal blood transfusion	**Modified Barthel Index**	10 days after surgery	No difference
				**New Mobility Score**	10 days after surgery	No difference
				**Ambulation score**	10 days after surgery	No difference
				Transfer independence	10 days after surgery	No difference
				Walking independence	10 days after surgery	No difference
				Mortality, per protocol	30-day	**Worse with restrictive**
				Mortality	90-day	No difference
				Leukocyte counts	30 days post-operatively	No difference
				CRP concentration	30 days post-operatively	No difference
				Infection	10 days post-operatively	No difference
				Complications	10 days post-operatively	No difference
				Modified Barthel Index	Day 30 to 1 year post-operatively	**Better with liberal**
				Depression	Day 30 post-operatively	No difference
					1 year post-operatively	No difference
Hempenius, 2013^29^	Pre & Post		Standard care	**Postoperative delirium**	10 days after surgery	No difference
		Care Protocol:		Severity of delirium	10 days after surgery	No difference
				Complications, >1	10 days after surgery	No difference
		Individualized geriatric care plan		Mortality	In-hospital	No difference
				SF-36 score	Discharge	No difference
				Care dependency	Assessed at discharge	No difference
				Return to an independent living situation	Assessed at discharge	**Worse with intervention**
				Additional care at home	Assessed at discharge	No difference
				Length of stay	In-hospital	No difference
Hoogeboom, 2010^30^	Pre	Exercise	Standard care	Osteoarthritis Outcome Score	Week before surgery	No difference
				Longitudinal Aging Study Amsterdam Physical Activity Questionnaire	Week before surgery	No difference
				Physical Working Capacity	Week before surgery	No difference
				6-MWT	Week before surgery	No difference
				Timed Up & Go Test	Week before surgery	No difference
				Chair Rise Time	Week before surgery	No difference
				Grip Strength	Week before surgery	No difference
				Time needed to functional independence	In-hospital	No difference
				Patient-Specific Complaints Questionnaire	Week before surgery	No difference
				Length of stay	In-hospital	No difference
Indrakusuma, 2014^25^	Pre	Care Protocol:	Standard care	**Mortality**	30 days post-operatively	No difference
		Nutrition supplements		**Postoperative delirium**	Not reported	No difference
		Cardiology consult		Postoperative complications	Not reported	No difference
		Blood transfusion		**Length of stay**	In-hospital	No difference
		Haloperidol prophylaxis				
Molino-Lova, 2011^34^	Post	Exercise	Usual aerobic exercise	Short Physical Performance Battery	1 year	**Better with intervention**
Oosting et al, 2012^31^	Pre	Exercise	Standard care	Timed Up & Go Test	6 weeks post-discharge	No difference
				6-MWT	6 weeks post-discharge	**Better with intervention**
				Chair Rise Time	6 weeks post-discharge	**Better with intervetion**
				Hip disability and Osteoarthritis Outcome Score	6 weeks post-discharge	No difference
				Longitudinal Aging Study Amsterdam Physical Activity Questionnaire	6 weeks post-discharge	No difference
				Pain	6 weeks post-discharge	No difference
				Patient Specific Complaints Questionnaire	6 weeks post-discharge	No difference
Opasich et al, 2010^32^	Post	Exercise	Traditional physiotherapy program	*Nursing needs	At discharge	**Better with intervention**
				Balance Performance Oriented Mobility Assessment	At discharge	**Better with intervention**
				Timed Up & Go Test	At discharge	**Better with intervention**
				Arm Curl	At discharge	**Better with intervention**
				Chair Stand	At discharge	**Better with intervention**
				6-MWT	At discharge	No difference
				Health related quality of life	At discharge	No difference
				Length of Stay	In-hospital	**Shorter with intervention**

* Primary outcome not specified in study. 6-MWT: 6 minute walk test; ADL: activities of daily living; CRP: c-reactive protein; SF: short form

**Bolded and underlined text** = Primary outcomes

**Bolded outcomes** reached statistical significance

#### Exercise interventions

Two studies evaluated the impact of preoperative exercise programs for elective total hip arthroplasty patients.[25,26] Participants in both trials were satisfied with the interventions, and both studies found positive impacts of exercise on functional outcomes. No improvements in postoperative function were noted.[25] Three studies evaluated postoperative exercise interventions, two in cardiac surgery and one after hip fracture surgery.[27–29] All three studies found positive impacts of the exercise intervention on functional outcomes, while in the lowest risk of bias study, the exercise intervention significantly improved quality of life outcomes.(28) Detailed description of the exercise interventions is provided in the Table C in S2 File, while a summary of evidence using the GRADE Framework[30] is provided in Table 3.

**Table 3:** GRADE summary of evidence.

Population-People with frailty having surgery						
Intervention-Exercise therapy						
Control-No or non-standardized exercise therapy						
Quality assessment						
Participants (studies)	Risk of bias	Inconsistency	Indirectness	Imprecision	Overall quality	Comment
**Postoperative function**						
503 (4)	Moderate	Low	No serious indirectness	No serious imprecision	Moderate^1^	Significant improvement in most physical performance measures in 3/4 studies
**Postoperative health related quality of life**						
314 (2)	Serious	Moderate	No serious indirectness	No serious imprecision	Low^2^	Significant improvement in physical and mental health in a randomized trial
**Postoperative length of stay**						
245 (2)	Moderate	Moderate	No serious indirectness	Moderate imprecision	Very low^3^	Decreased length of stay in larger observational study; none in small pilot randomized trial

1. Downgraded as not all studies showed improvement, and 1 was non-randomized

2. Downgraded due to unclear allocation concealment and blinding in RCT, no effect in observational study

3. Downgraded due to inconsistency, positive effect was from a high risk of bias observational study

#### Multicomponent geriatric care protocols

Prior to elective colorectal surgery, geriatric assessment to guide perioperative care planning was associated with decreased length of hospital stay, however no differences in primary or other outcomes were identified.[31]

Geriatric-specific multicomponent interventions were tested in three observational studies, two of which included general surgery patients,[24,32] and the third which included a mix of surgical specialties.[33] Following elective general surgery, institution of a modified hospital elder life program (a formal evidence-based program to optimize care of older patients in hospital[34]) was associated with a lower rate of frailty at hospital discharge.[32] Following institution of a team-based complex geriatric intervention for a mixed surgical population, there was no significant difference in primary or most secondary outcomes.[33] A structured geriatric rehabilitation program after laparoscopic cholecystectomy was associated with improvements in functional, nutritional and cognitive outcomes.[24]

A single RCT evaluated a geriatric care protocol with pre- and postoperative components in elective cancer surgery.[35] The authors found that the individuals in the intervention group, who underwent preoperative geriatric assessment, individualized delirium prevention plans, daily geriatric nurse liaison while in hospital and consultative treatment advice experienced similar rates of delirium and other outcomes compared to those who received standard care.

Poor protocol adherence was noted in two of five multicomponent studies, [31,33] while another multicomponent study reported difficulties with the complexity of applying and measuring adherence to the study’s specific protocol components.[35] Details of each multicomponent intervention and control group care are provided in theTable D in S2 File.

#### Transfusion trigger

Following hip fracture surgery, one study of a restrictive vs. liberal red blood cell transfusion strategy found no differences in mortality, quality of life, functional outcomes, or infectious complications between arms. The authors did report an increase in 30-day mortality in the restrictive arm per their secondary per protocol analysis, however, there were an equal number of protocol violations in both study arms, and at 90 days there was no difference in mortality, even when analyzed per protocol.[36–38]

### Risk of bias

**Fig 3:**
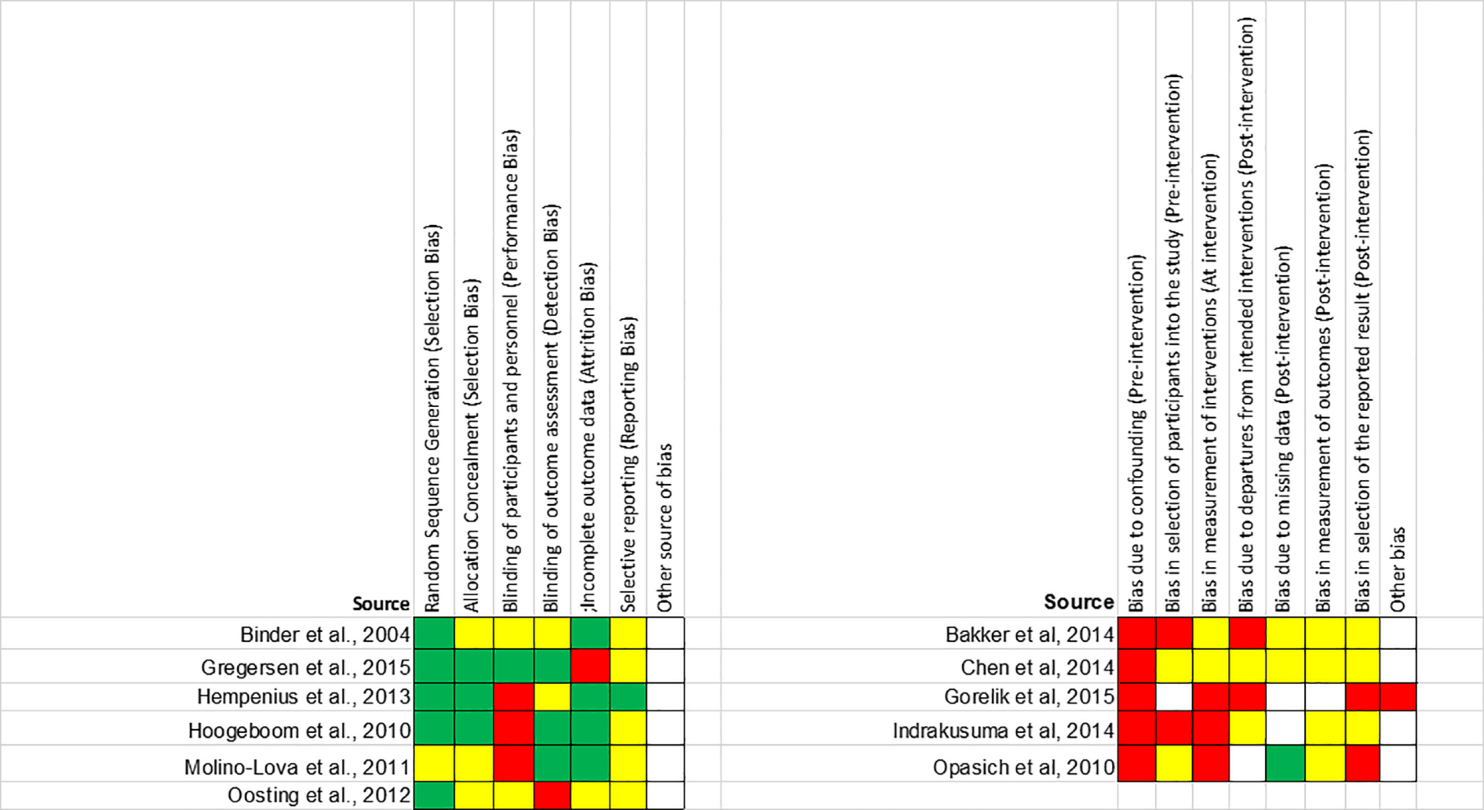
Risk of bias assessment. Green represents low risk of bias, yellow moderate risk of bias and red high risk of bias. For domains with white squares, risk of bias was unclear.

Two RCTs were assessed as moderate risk of bias; all others were at high risk of bias. Performance bias related to blinding of participants, and selective outcome reporting were the domains most often rated as moderate to high risk of bias. All observational studies were at high risk of bias, and in particular suffered from confounding bias (Fig 3).

### Outcomes

Based on the Triple Aim Framework, all studies reported at least one health outcome, eight studies reported a patient experience outcome, and cost outcomes were reported in four studies. Seven studies specified a primary outcome, while four studies reported on multiple outcomes without specifying a primary outcome of interest. A formal meta-analysis was not possible due to the heterogeneity of study designs, interventions and outcomes.

## Discussion

A substantial proportion of postoperative adverse events in older surgical patients are attributable to the presence of frailty. However, despite a marked increase in the epidemiological literature describing associations between frailty and adverse postoperative outcomes, we identified only eleven studies that tested interventions in populations of frail patients having surgery. Although six out of eleven studies identified were RCTs, only two of eleven studies were not at high risk of bias. The small number of studies identified, and the high risk of bias present in most studies, highlights a substantial knowledge gap in surgery and perioperative medicine. There is an urgent need for the development and testing of new interventions to improve the outcomes of frail people having surgery, as well as large, multicenter RCTs at low risk of bias to evaluate promising interventions, such as perioperative exercise therapy in the frail elderly.

Even with a broad search strategy and no specific limitations on the frailty definitions eligible for inclusion, or the intervention types considered, our systematic review identified only eleven studies that tested perioperative interventions in frail patients. In part, this does reflect our protocol’s requirement that a frailty definition be used. This lead to exclusion of studies of hip fracture patients which did not include specified frailty definitions. While some consider a hip fracture to be frailty-defining, not all older hip fracture patients are found to be frail when frailty criteria are applied.[39,40] Furthermore, geriatric-specific interventions, such as the Proactive care of Older People having Surgery,[41] have been tested in higher-risk older surgical patients, and show promising impacts on outcomes. While some included patients in this study were likely frail, the frailty definition requirement of our protocol excluded this study as our aim was to identify evidence that could be generalized specifically to frail older people, who are a unique stratum of the population of older people having surgery.

Only one conference abstract and seven study protocols were identified, suggesting that the small pool of published studies identified is not about to increase substantially. Given our study’s strengths, including pre-registration of our study protocol, and adherence to best practice methodologies (such as duplicate handling of all stages of the review, grey literature searches, and hand searching of study reference lists) the paucity of identified studies underlies an urgent call for a transition from the current focus of describing the epidemiology of perioperative frailty to efforts to prospectively address the risk of frailty in patients having surgery. These efforts should also include younger people with frailty, who were not represented in any of our included studies. Moving forward, investigators will need to study interventions which address the factors that we currently understand to contribute to the adverse outcome burden experienced by frail people having surgery. Although not yet comprehensively understood, these factors include vulnerability to intrinsic and extrinsic stressors, decreased cognitive reserve, and dysregulation of immune and inflammatory mechanisms.[8,42] To support this move, investigators must commit to performing low risk of bias randomized trials (with a particular focus on improved blinding, allocation concealment, and outcome pre-specification). Where randomized trials aren’t indicated or feasible, improved observational study methodologies, such as interrupted time series analyses or other quasi-randomized designs should be considered in place of controlled before after studies. Furthermore, no intraoperative interventions, such as comparison of specific surgical techniques for frail patients, have been reported.

Despite the limitations present in our included studies, including the heterogeneity of frailty definitions, intervention types, surgical populations and outcome measures that precluded meta-analysis and formal assessment of publication bias, and the substantial risk of bias across studies, our findings do provide important insights to guide the improvement of outcomes for frail surgical patients. Perioperative exercise therapy appears to be a promising intervention to improve function and quality of life, and we identified consistent barriers in studies which attempted to implement and test multicomponent geriatric-specific care protocols. These insights are discussed in the following paragraphs.

### Exercise therapy

In all five studies that evaluated perioperative exercise therapy, the intervention was positively associated with improved function, quality of life, or both. Findings from two RCTs[28,29] and one before after study[27] found that postoperative exercise therapy in cardiac and orthopedic surgery populations improved outcomes. Therefore, while confirmation of these findings in a high quality multicenter RCT would be preferable, we suggest that the consistent directional association that was generalized across surgical populations supports inclusion of postoperative exercise therapy in the perioperative care of frail surgical patients. Preoperative exercise therapy requires a more thorough evaluation in future studies, as the two small RCTs that we identified primarily evaluated changes in *preoperative* function. Furthermore, neither was designed or powered to adequately evaluate the impact of preoperative exercise on postoperative functional recovery or other outcomes. Therefore, a high quality RCT of preoperative exercise in frail older patients that is properly powered and designed to evaluate meaningful differences in long-term postoperative outcomes is needed. Studies that include pre- and postoperative exercise interventions should also be considered.

### Multicomponent geriatric interventions

Despite the positive impact on outcomes of multicomponent interventions such as orthogeriatric care in older hip fracture surgery patients (who are often frail),[43,44] the five studies of multicomponent geriatric-focused care protocols included in our study did not demonstrate consistent improvements in outcomes. In fact, only one study clearly found a positive association between protocol implementation and the primary study outcome. Chen et. al.,[32] who implemented a modified version of a pre-existing evidence-based intervention found that protocol implementation was associated with improved frailty status at hospital discharge. Interestingly, the authors describe use of standardized training materials and a specially trained nurse-educator to implement and support compliance with the protocol. In contrast, the three studies that clearly failed to demonstrate an improvement in their primary outcome all reported issues with protocol implementation and non-compliance[31,33,35] (methodological and reporting limitations from the fifth care protocol study precluded clear interpretation of its findings[24]). Therefore, in addition to ensuring that interventions included in geriatric-focused multicomponent interventions for frail surgical patients are evidence based, there is also a need to consider the feasibility of each intervention, as well as the clinical context, to support success.

### Choice of frailty instrument

Although the adverse outcome effect of frailty appears to generalize across different frailty instruments, the generalizability of current and future interventional study findings will be limited in the absence of efforts to standardize, or at least limit, the number of different frailty instruments used in perioperative research. Consistent with previous reports from other areas of frailty research (such as non-surgical frailty,[45] or non-interventional studies of perioperative frailty[8,17]), we identified substantial heterogeneity in the instruments used to define frailty. In the eleven included studies, eight different frailty definitions were used. Although the modified Fried Index,[46] a phenotypic approach to frailty diagnosis, is recommended by practice guidelines,[47] only one recent publication has compared the predictive performance of different frailty instruments to inform the choice of an appropriate perioperative tool.[48] Further comparative research and consensus building is needed. Without consensus, clinicians will be limited in their ability to apply study findings to people with frailty having surgery, and future efforts in knowledge synthesis will be significantly hindered by heterogeneity in frailty definitions.

### Outcomes reported

The variety of outcomes evaluated in identified studies is both promising, and a cause for concern. Encouragingly, studies did not focus only on traditional outcomes such as morbidity, mortality and length of stay, but also evaluated patient experience, function, and quality of life. In fact, all three domains of the IHI Triple Aim were well-represented. However, the heterogeneity in outcome measures also draws attention to the lack of agreed upon core outcomes for the frail elderly in general, or frail people having surgery more specifically. Engagement of processes such as the Core Outcome Measures in Effectiveness Trials (COMET) initiative[49] to define a minimum set of key outcomes for frail surgical patients is needed.

## Conclusions

Only a small number of studies exist which investigate the impact of perioperative interventions on outcomes in frail surgical patients. Although exercise interventions appear to show promise in improving functional and quality of life outcomes, further studies are needed to address methodological limitations identified in the existing literature. Development of multicomponent geriatric care protocols require consideration of anticipated efficacy as well as feasibility to support effective implementation. Significant efforts are needed to develop evidence-informed interventions to improve the outcomes of our growing frail surgical population, and to evaluate these interventions in low risk of bias studies.

## Supporting information

S1 File PRISMA 2009 checklist for reporting of systematic reviews. (DOC)

S2 File Table A- Search strategies for included databases; Table B—Conference abstracts and study protocols identified; Table C—Description of exercise interventions and control conditions; Table D- Description of multicomponent geriatric care protocols and control conditions.
(DOCX)
