# Supplementary material for: Interventions to improve the outcomes of frail people having surgery: A systematic review
Source: PLoS One. 2017 Dec 29;12(12):e0190071. doi: 10.1371/journal.pone.0190071 (PMC5747432; doi:10.1371/journal.pone.0190071)
Supplement: S2 File — Table A- Search strategies for included databases; Table B—Conference abstracts and study protocols identified; Table C—Description of exercise interventions and control conditions; Table D- Description of multicomponent geriatric care protocols and control conditions. (DOCX) [file pone.0190071.s002.docx]

**S2 File**

**Table A – Search strategies for each included database**

**Database: Epub Ahead of Print, In-Process & Other Non-Indexed Citations, Ovid MEDLINE(R) Daily and Ovid MEDLINE(R)**

--------------------------------------------------------------------------------

1 Frail Elderly/

2 frail*.tw,kw.

3 1 or 2

4 perioperative period/ or intraoperative period/ or postoperative period/ or preoperative period/

5 Surgical Procedures, Operative/ or Elective Surgical Procedures/

6 (perioperative* or peri-operative*).tw,kw.

7 (preoperative* or pre-operative*).tw,kw.

8 (postoperative* or post-operative*).tw,kw.

9 (surgery or surgical).tw,kw.

10 exp Hip Fractures/su or exp Arthroplasty/

11 or/4-10

12 3 and 11

**Database: Embase Classic+Embase**

--------------------------------------------------------------------------------

1 frail elderly/

2 frail*.tw.

3 1 or 2

4 *perioperative period/ or *intraoperative period/ or *postoperative period/ or *preoperative period/ or preoperative evaluation/

5 (perioperative* or peri-operative*).tw.

6 (preoperative* or pre-operative*).tw.

7 (postoperative* or post-operative*).tw.

8 exp *surgery/ or surgical patient/

9 (surgery or surgical).ti.

10 exp arthroplasty/ or hip fracture/su or femur neck fracture/su

11 or/4-10

12 3 and 11

**Cochrane**

#1 MeSH descriptor: [Frail Elderly] explode all trees

#2 frail*:ti,ab,kw (Word variations have been searched)

#3 #1 or #2

#4 MeSH descriptor: [Perioperative Period] explode all trees

#5 MeSH descriptor: [Surgical Procedures, Operative] this term only

#6 MeSH descriptor: [Elective Surgical Procedures] this term only

#7 (perioperative* or peri-operative*):ti,ab,kw (Word variations have been searched)

#8 (preoperative* or pre-operative*):ti,ab,kw (Word variations have been searched)

#9 (postoperative* or post-operative*):ti,ab,kw (Word variations have been searched)

#10 (surgery or surgical):ti,ab,kw (Word variations have been searched)

#11 #4 or #5 or #6 or #7 or #9 or #9 or #10

**#12 #3 and #11**

**CINAHL**

| **#** | **Query** |
| --- | --- |
| **S21** | **S3 AND S20** |
| S20 | S4 OR S5 OR S6 OR S7 OR S8 OR S9 |
| S9 | TI ( (surgery or surgical) ) OR AB ( (surgery or surgical) ) |
| S8 | (MH "Surgery, Operative+") |
| S7 | TI ( (postoperative* or post-operative*) ) OR AB ( (postoperative* or post-operative*) ) |
| S6 | TI ( (preoperative* or pre-operative*) ) OR AB ( (preoperative* or pre-operative*) ) |
| S5 | TI ( (perioperative* or peri-operative*) ) OR AB ( (perioperative* or peri-operative* ) |
| S4 | (MH "Perioperative Care+") OR (MH "Preoperative Care+") OR (MH "Postoperative Care+") OR (MH "Intraoperative Care+") |
| S3 | S2 OR S2 |
| S2 | TI frail* OR AB frail* |
| S2 | (MH "Frailty Syndrome") |

**Table B – Grey literature and ClinicalTrilas.gov search results summary**

Summaries provided in PICO format (**P**opulation, **I**ntervention, **C**ontrol, **O**utcome)

**Conference Abstracts Identified**

Lococo F, Margaritora S, Nachira D, et. al. Clinical effect of bovine pericardial strips on air leak after stapled pulmonary resection in ‘frail’ patients: early results. European Surgical Research 2012, 49: 186

P-Frail patients, pulmonary resection planned

I-bovine pericardial strips added to pulmonary resection closure

C-Standard closure

O-Air leak

Results-17.8% reduction in air leak in intervention group

**ClinicalTrials.gov protocols**

Clinical Intervention in Frail Older People (FRAILCLINIC). NCT02643069. Currently recruiting

P-Frail, >=75, surgery, oncology or medical patients

I- Geriatric care in-hospital and after discharge

C-Usual care

O-Barthel Index for Activities of Daily Living

FitHips: Getting Fit for Hip Replacement (FitHips). NCT02885337. Currently recruiting

P-frail or pre-frail, >=65, unilateral hip replacement with >=3 month wait expected

I-Multicomponent care (exercise, cognitive, nutrition, vitamin, and med review interventions)

C-Usual care

O-Feasibility

Geriatric Intervention in Frail Elderly Patients With Colorectal Cancer. NCT01321658. Completed (no results posted)

P-Frail, >=65 years, surgery for colorectal surgery

I-Individualized care plan based on comprehensive geriatric assessment

C-Standard care

O-Postoperative complications

Intervention for PreFrailty and Frailty in Thoracic Surgery Patients. NCT02522533. Currently recruiting

P-Frail or prefrail

I-Exercise therapy

C-Single arm study

O-Reduction in frailty

Off Pump Versus On Pump Coronary Artery Bypass Grafting in Frailty Patients (FRAGILE) (FRAGILE). NCT02338947. Not yet open for recruitment

P-Frail, >=65 years

I-Off pump cardiac surgery

C-On pump cardiac surgery

O-cardiac and cerebrovascular complications

Preoperative Rehabilitation for Reduction of Hospitalization After Coronary Bypass and Valvular Surgery. (PREHAB). NCT02219815. Currently recruiting

P-Frail, >=60, cardiac surgery

I-Preoperative exercise therapy

C-Standard Care

O-proportion of patients with length of stay > 7 days

The Prehabilitation Study: Exercise Before Surgery to Improve Patient Function in People. NCT02934230. Not yet open for recruitment

P-Frail, >=65 years, elective cancer surgery

I-Preoperative exercise therapy

C-Standard care

O- 6-minute walk test

**Table C** – Description of exercise interventions and control conditions

|  | Intervention description | Control condition |
| --- | --- | --- |
| Binder et al, 2004 | 2-phase exercise program (3 months per phase). In each phase there were 3 sessions per week; sessions were 45-90 minutes long. Phase 1 was physical therapist-directed protocol that consisted of 22 exercises that were advanced in difficulty as participant's physical performance improved. Phase 2 included the addition of progressive weighted resistance exercises. Again, difficulty was added as participants progressed | Low-intensity home-based program with 9 exercises following a 1 hour introductory session. Monthly, control participants could attend a 1 hour refresher. 3 home-based sessions per week were prescribed, and weekly checkins were performed by phone. |
| Hoogeboom et al, 2010 | Twice weekly supervised exercise sessions for 3-6 weeks before surgery. Each 60 minute session including a 5 minute walking warm-up, 10-20 sets of leg press, and 20-30 minutes on a cycle ergometer. Except for warmup, exercises were performed at a moderate to high intensity. Participants were encouraged to increase their activity levels in their day-to-day life | A single group-based education session on early mobilization, surgery and anesthesia techniques, restricted movements, benefits of activity and proper use of crutches (1 week before surgery) |
| Molino-Lova et al, 2011 | Aerobic exercise prescription plus 2 sessions teaching exercises for strength, balance, flexibility, and coordination. Additional exercises included upper and lower limb strengthening exercises, stretching, tandem and semi-tandem balance walking, upper and lower limb coordination exercises. | Aerobic exercise prescription |
| Oosting et al, 2012 | Two, 30-minute sessions for 3-6 weeks before surgery to improve functional activities and walking capacity. Exercises were progressively advanced. Additionally, participants were asked to walk for 30 minutes per days 4 times per week. Intensity target was 11-13 on the Borg Scale | Single group session with a physiotherapist 3 weeks before surgery. This involved teaching about crutches and exercises that would be done after surgery. |
| Opasich et al, 2010 | Personalized postoperative training based on frailty stratification. Severely frail patients received individualized training and nursing care until adequate balance was achieved to safely do exercise. Moderately frail patients did daily sessions with strength and flexibility exercises with incremental increases in resistance. | Standard physiotherapy protocol with calisthenics and cycle/treadmill sessions. |

**Table D** – Description of multicomponent geriatric care protocols and control conditions

| **Source** | **Intervention Timing** | **Intervention** | **Control Group Intervention** | **Intervention Details** |
| --- | --- | --- | --- | --- |
| Bakker et al, 2014 | Pre- and Post-operative | Care Protocol: | Standard care: Not described | **First screening by nurses**: A nurse judges a patient to be potentially frail when the patient is at risk of delirium, malnutrition, physical decline, and/or falling, on/before the day of admission. The used instrument is obliged to be used by all Dutch hospitals for all patients aged R70 years and therefore chosen as an appropriate screening instrument to integrate with usual care. |
|  |  | Orientation |  | **Second screening by geriatrics nurse:** Clinical judgment of frailty by a geriatrics nurse based on the nursing and medical file (including medication) and a brief interview with the patient. To create a uniform procedure which is feasible and efficient in practice, the way in which the clinical judgments are made are discussed during the monthly meetings with the intervention team. |
|  |  | Mobilization |  | **Medication review:** The geriatrician (or resident) critically evaluates the following details: medical information and medication use obtained from the medical file; if possible, information regarding the use of medications by the patient before admission; information from the primary care physician when the indication for a medication is unclear. The evaluation of medication is performed by following pre-set steps and criteria. |
|  |  | Day program activities |  | **CareWell plan**: Contains recommendations about care and well-being provided by the CareWell team, communicated both verbally and written, to which nurses and physicians should adhere and are responsible. These recommendations regarding health, well-being, support from trained volunteers, medication, after care and goal attainment are categorized under the following headings: somatic problems, physical functioning, social environment, psychosocial functioning, and communication. Recommendations follow findings from a conducted interview with the patient by a geriatrics nurse, partially based on the EasyCare method. |
|  |  | Physiotherapy consult |  | **Follow-up during admission and update of the CareWell plan on discharge**: Follow-up comprises checking for adherence and evaluating the recommendations of the CareWell team, from nursing files and multidisciplinary meetings. Update of the CareWell plan 1 day before discharge consists of the formulation of geriatric information to be added to the discharge letter to the primary care physician. |
|  |  | Dietitian consult |  | **Medical history by proxy**: An interview conducted with a close relative of the patient or another care professional to obtain information regarding the patient’s health situation when additional and/or reliable information is needed. |
|  |  | Discharge planning |  | **Comprehensive geriatric assessment**: An extensive multidisciplinary clinical geriatric assessment that is used to create a coordinated and integrated care plan for an individual highly frail patient, performed by a geriatrician (or resident). |
|  |  | Medication review |  | **Multidisciplinary meeting**: A weekly meeting within the department with geriatric input, in which several care professionals are involved, including the geriatrics nurse and geriatrician to adjust the medical and nursing policies to the individual needs and wishes of a frail elderly patient. |
|  |  | CGA by geriatrician |  | **Stimulation of cognitive and physical activities by trained volunteers**: CWH volunteers work on the basis of an instruction protocol, as part of the CareWell plan, which contains concise information regarding (approaching) the patient and the four programs orientation, therapeutic activities, physical activities, and nutrition; is indicated by a geriatrics nurse; is created daily and adapted whenever necessary by a CWH volunteers’ coordinator; and is to be followed twice daily on weekdays and once daily during the weekend. |
|  |  |  |  | **Education of nurses and physicians:** The geriatrics team provides educational sessions on group level on all abovementioned intervention parts as well as continuous coaching-on-the-job. |
| Chen et al, 2014 | Post-operative | Care Protocol: | Standard care: Care by physicians and nurses (same clinicians in pre- and post-implementations phase); referral to dieticians and physical therapists as needed. | The mHELP comprised three mHELP interventions (early mobilization, oral and nutritional assistance, and orientating communication). Participants in the intervention group received mHELP in addition to usual care as soon as they arrived on the surgical inpatient ward. Using standardized mHELP manuals, a registered nurse with more than 2 years of experience in medical–surgical nursing was trained as the HELP nurse. This 2-month on-site training included review of manuals and weekly individual mentorship. This same HELP nurse, who was blinded to the study hypotheses and did not serve as an outcome assessor, provided all three modified HELP interventions three times daily during the study. |
|  |  | Early mobilization |  |  |
|  |  | Oral and nutritional assistance |  | In the mobilization intervention, this HELP nurse assisted participants in completing physical activities, including range-of-motion exercises in bed, sitting up, riding a stationary bicycle by hand or foot, standing, or ambulating, according to their capacity. While performing activities, the HELP nurse deliberately engaged participants in orienting communication (e.g., recalling and discussing topics that interested them, such as events on the operative day), reinforcing orienting content. Daily oral care (tooth brushing and range-of-motion exercises for lips, tongue, and jaw) and diet education for postsurgical intake were also provided. Approximately 30 to 45 minutes per day was added to the care of each participant as a result of the mHELP protocol. |
|  |  | Orientating communication |  |  |
| Gorelik et al, 2015 | Post-operative | Care Protocol: | Standard care: Not described | The main reasons defining severity (minor, medium, heavy) of frailty were determined, non-medicinal and medicinal methods and means of rehabilitation, aimed on correction of main geriatric syndromes in home conditions involving team practice were used. The team included: district doctor, surgeon, dietitian, rehabilitologist, nurse, doctor in exercise therapy, social worker, clinical psychologist. The major non-medicinal methods and means of rehabilitation, administered on ambulatory-clinical stage of rehabilitation: use of compensating devices, self-service training, use of exercise therapy, labor therapy, rational psychotherapy, diet correction, creation of therapeutic conditions for the patient. Correction of treatment and efficincy control were carried out by the district doctor once per 3 months on the basis of special geriatric exam. Frequency of examinations conducted by other members of the team is determined by the necessity of their advisory support. |
|  |  |  |  |  |
|  |  | Rehabilitation |  |  |
|  |  | Nutrition support |  |  |
|  |  | Psychotherapy |  |  |
|  |  | Home care for some |  |  |
| Hempenius et al, 2013 | Pre- and Post-operative | Care Protocol: | Standard care: additional geriatric care only provided at request of treating physician | The multicomponent intervention focused on best supportive care and the prevention of delirium. Patients in the intervention group were assessed preoperatively by a geriatric team and monitored during their hospital stay. As the three participating centres are heterogeneous and this could cause variance in how the intervention was conducted, checklists were used to standardize the intervention as much as possible. The geriatric team was supervised by a geriatrician, and helped devise the individual care plan. The preoperative comprehensive geriatric assessment by a geriatrician consisted of a medical history, physical examination and follow-up examinations on indication. In order to standardize this consultation a checklist was composed based on expert opinion. This checklist contained items concerning medication, co-morbidities, loss of vision and hearing, nutrition, mobility, depression, incontinence and cognitive, social and instrumental functioning (instrumental Activities of Daily Life. An individual treatment plan was drawn up paying specific attention to patient-related risk factors for delirium, namely, cognitive impairment, visual impairment, hearing impairment, malnutrition and impaired mobility. Preventive pharmacological measures were an optional but non-imperative part of the intervention protocol. During their hospital stay, the patients in the intervention group were assessed daily by a geriatric nurse. A daily checklist was used to ensure the uniformity of the geriatric intervention in the participating centres. This checklist consisted of nine items: orientation, mobility, anxiety, senses, pain, sleep, intake, defecation and infection. If a problem concerning one of these was encountered, the geriatric nurse or geriatrician contacted the treatment team to discuss the proposed intervention and establish a treatment plan, checking daily to determine whether the advice had been followed. |
|  |  |  |  |  |
|  |  | Individual care plan as devised by the geriatric team. There was heterogeneity in interventions amongst the 3 participating hospitals, but a checklist was used in an attempt to address the differences. |  |  |
|  |  |  |  |  |
|  |  | Checklist contained items concerning medication, co-morbidities, loss of vision and hearing, nutrition, mobility, depression, incontinence and cognitive, social and ADLs. |  |  |
|  |  |  |  |  |
|  |  |  |  |  |
|  |  |  |  |  |
|  |  |  |  |  |
| Indrakusuma et al, 2014 | Pre-operative | Care Protocol: | Standard care: Not described | Vitamin supplementation |
|  |  | Vitamin and dietary supplementation |  | Preoperative dietary supplements |
|  |  | Cardiology consult |  | Preoperative consult with cardiologist |
|  |  | Blood transfusion |  | Preoperative transfusion |
|  |  | Haloperidol prophylaxis for those at risk for post-operative delirium |  | Haloperidol prophylaxis for those at risk of post-operative delirium |
